# Supplementary material for: Surveys of Knowledge and Awareness of Plastic Pollution and Risk Reduction Behavior in the General Population: A Systematic Review
Source: Int J Environ Res Public Health. 2025 Jan 27;22(2):177. doi: 10.3390/ijerph22020177 (PMC11855307; doi:10.3390/ijerph22020177)
Supplement: Supplementary file 1 [file ijerph-22-00177-s001.zip › Supplementary material/Table S2.pdf]

**Table S2: Full search strategies used for each database**

| Database | Search string |
| --- | --- |
| PubMed | ("Plastics"[MeSH Terms] OR "Microplastics"[MeSH Terms] OR "biodegradable plastics"[MeSH Terms] OR "Plastics"[Title] OR "biodegradable plastics"[Title] OR "microplastic*"[Title] OR "micro plastic*"[Title] OR "nanoplastic*"[Title] OR "nano plastic*"[Title] OR "plastic material"[Title] OR "plastic pollution"[Title] OR "plastic waste"[Title] OR "plastic litter"[Title] OR "plastic exposure"[Title]) AND ("Surveys and Questionnaires"[MeSH Terms] OR "Survey"[Title/Abstract] OR "questionnaire"[Title/Abstract] OR "perception*"[Title/Abstract] OR "perceive*"[Title/Abstract] OR "perspective*"[Title/Abstract] OR "opinion*"[Title/Abstract] OR "view*"[Title/Abstract] OR "behavio*"[Title/Abstract] OR "feeling*"[Title/Abstract] OR "attitude*"[Title/Abstract] OR "knowledge"[Title/Abstract] OR "awareness"[Title/Abstract]) AND ("Public"[Title/Abstract] OR "people"[Title/Abstract] OR "citizens"[Title/Abstract] OR "population"[Title/Abstract] OR "residents"[Title/Abstract] OR "inhabitants"[Title/Abstract] OR "consumers"[Title/Abstract]) |
| EmBase | 'plastic'/exp OR 'microplastic'/exp OR 'nanoplastic'/exp OR 'biodegradable plastic'/exp OR plastics:ti OR microplastics:ti OR nanoplastics:ti OR 'biodegradable plastics':ti OR microplastic*:ti OR 'micro plastic*':ti OR nanoplastic*:ti OR 'nano plastic*':ti OR 'plastic material':ti OR 'plastic pollution':ti OR 'plastic waste':ti OR 'plastic litter':ti OR 'plastic exposure':ti) AND ('questionnaire'/exp OR survey:ab,ti OR questionnaire:ab,ti OR perception*:ab,ti OR perceive*:ab,ti OR perspective*:ab,ti OR opinion*:ab,ti OR view*:ab,ti OR behavio*:ab,ti OR feeling*:ab,ti OR attitude*:ab,ti OR knowledge:ab,ti OR awareness:ab,ti) AND (public:ab,ti OR people:ab,ti OR citizens:ab,ti OR population:ab,ti OR residents:ab,ti OR inhabitants:ab,ti OR consumers:ab,ti) |
