# Supplementary material for: Surveys of Knowledge and Awareness of Plastic Pollution and Risk Reduction Behavior in the General Population: A Systematic Review
Source: Int J Environ Res Public Health. 2025 Jan 27;22(2):177. doi: 10.3390/ijerph22020177 (PMC11855307; doi:10.3390/ijerph22020177)
Supplement: Supplementary file 1 [file ijerph-22-00177-s001.zip › Supplementary material/Table S3.pdf]

Table S3: Survey quality evaluation tool

Main question / Sub-question		Weight of sub-question:
**1 Was a clear research question posed?**		
	Does the research question or objective specify clearly the type of respondents?	0.33
	Does the research question or objective specify clearly the topic of interest?	0.33
	Does the research question or objective specify clearly the primary and secondary research questions to be addressed?	0.33
**2 Was the target population defined, and was the sample representative of the population?**		
	Was the population of interest specified?	0.5
	Was the sampling frame specified?	0.5
**3 Was a systematic approach used to develop the questionnaire?**		
	Item generation and reduction: Did the authors report how items were generated?	0.25
	Item generation and reduction: Did the authors report how items ultimately reduced?	0.25
	Questionnaire formatting: Did the authors specify how questionnaires were formatted?	0.25
	Pretesting: Were individual questions within the questionnaire pretested?	0.25
**4 Was the questionnaire tested?**		
	Pilot testing: Was the entire questionnaire pilot-tested?	0.5
	Clinimetric testing: Were any clinimetric properties (face validity or clinical sensibility testing, content validity, inter- or intra-rater reliability) evaluated and reported?	0.5
**5 Were questionnaires administered in a manner that limited both response and nonresponse bias?**		
	Was the method of questionnaire administration appropriate for the research objective or question posed?	0.33
	Were additional details regarding prenotification, use of a cover letter provided?	0.33
	Were additional details regarding prenotification, an incentive for questionnaire completion provided?	0.33
**6 Was the response rate reported, and were strategies used to optimize the response rate?**		
	Was the response rate reported (alternatively, were techniques used to assess nonresponse bias)?	0.25
	Was the response rate defined?	0.25
	Were strategies used to enhance the response rate (including sending of reminders)?	0.25
	Was the sample size justified?	0.25
**7 Were the results clearly and transparently reported?**		
	Does the survey report address the research question(s) posed or the survey objectives?	0.125
	Were methods for handling missing data reported?	0.125
	Were demographic data of the survey respondents provided?	0.125
	Were the analytical methods clear?	0.125
	Were the results succinctly summarized?	0.125
	Did the authors' interpretation of the results align with the data presented?	0.125
	Were the implications of the results stated?	0.125
	Was the questionnaire provided in its entirety (as an electronic appendix or in print)?	0.125
**TOTAL**		**7**

Note: Guide by Burns and Kho (doi: 10.1503/cmaj.140545) designed to help readers systematically appraise the quality of survey reports
