# Supplementary material for: Surveys of Knowledge and Awareness of Plastic Pollution and Risk Reduction Behavior in the General Population: A Systematic Review
Source: Int J Environ Res Public Health. 2025 Jan 27;22(2):177. doi: 10.3390/ijerph22020177 (PMC11855307; doi:10.3390/ijerph22020177)
Supplement: Supplementary file 1 [file ijerph-22-00177-s001.zip › Supplementary material/Table S4.pdf]

Table S4: Excluded studies with reasons for ineligibility

First author and year of publication	Title	Journal	Reason for exclusion
Elmosaad YM 2023	Self-Reported Household Waste Recycling and Segregation Practices among Families in Eastern Region of Saudi Arabia: A Cross-Sectional Study	International Journal of Environmental Research and Public Health	1. Focused on specific topic
Escario JJ 2020	The influence of environmental attitudes and perceived effectiveness on recycling, reducing, and reusing packaging materials in Spain	Waste Management	1. Focused on specific topic
Holmberg K 2023	Keep plastics on a tight leash: Swedish public opinion on plastic policies	Environmental Science & Policy	1. Focused on specific topic
Hu J 2024	Waste separation behavior with a new plastic category for the plastic resource circulation: Survey in Kansai, Japan.	Journal of environmental management	1. Focused on specific topic
Jahani A 2019	Iranian experiences in terms of consumption of disposable single- use plastics: Introduction to theoretical variables for developing environmental health promotion efforts.	Environmental toxicology and pharmacology	1. Focused on specific topic
Jia Q 2023	Understanding residents' behaviour intention of recycling plastic waste in a densely populated megacity of emerging economy.	Heliyon	1. Focused on specific topic
Khan F 2019	Understanding consumers' behavior intentions towards dealing with the plastic waste: Perspective of a developing country	Resources, Conservation and Recycling	1. Focused on specific topic
Kochanska E 2022	Global Ban on Plastic and What Next? Are Consumers Ready to Replace Plastic with the Second-Generation Bioplastic? Results of the Snowball Sample Consumer Research in China, Western and Eastern Europe, North America and Brazil.	International journal of environmental research and public health	1. Focused on specific topic
McDonald S 1998	Public participation in plastics recycling schemes	Resources, Conservation and Recycling	1. Focused on specific topic
Northen SL	From shops to bins: a case study of consumer attitudes and behaviours towards plastics in a UK	Sustainability science	1. Focused on specific

2023	coastal city.		topic
Roche Cerasi I 2021	Household plastic waste habits and attitudes: A pilot study in the city of Valencia.	Waste management & research : the journal of the International Solid Wastes and Public Cleansing Association, ISWA	1. Focused on specific topic
Roy D 2022	"If it is not made easy for me, I will just not bother". A qualitative exploration of the barriers and facilitators to recycling plastics.	PloS one	1. Focused on specific topic
Sharma P 2021	Assessment of awareness regarding health hazards of plastic chemicals and their warning label among a sample population of Varanasi City: A cross-sectional study	*Journal of Pharmacy and Bioallied Sciences*	1. Focused on specific topic
Willis B 2022	Towards Microplastic Reduction Within Institutions.	Water, air, and soil pollution	1. Focused on specific topic
Afroz R 2017	The knowledge, awareness, attitude and motivational analysis of plastic waste and household perspective in Malaysia.	Environmental science and pollution research international	2. Qualitative study
Felipe-Rodriguez M 2022	What does the public think about microplastics? Insights from an empirical analysis of mental models elicited through free associations.	Frontiers in psychology	2. Qualitative study
Henderson L 2020	Making sense of microplastics? Public understandings of plastic pollution.	Marine pollution bulletin	2. Qualitative study
Janzik R 2023	Exploring public risk perceptions of microplastics: Findings from a cross-national qualitative interview study among German and Italian citizens.	Risk analysis : an official publication of the Society for Risk Analysis	2. Qualitative study
Nguyen HV 2022	Intrinsic motivation for reducing single-use plastics: The compensation effects of basic psychological needs	Resources, Conservation and Recycling	2. Qualitative study
Oduro-Appiah K 2023	Using an extended model of the reasoned action approach to explore individual behavioral intentions regarding litter and plastic pollution prevention in a developing country.	Frontiers in psychology	2. Qualitative study
Phan TT 2023	Evaluating citizensâ€™ willingness to participate in hypothetical scenarios towards sustainable	Environmental Science & Policy	2. Qualitative study

	plastic waste management		
Aubin S 2022	Plastics in a circular economy: Mitigating the ambiguity of widely-used terms from stakeholders consultation	Environmental Science & Policy	3. Study objectives not relevant
Liu TK 2023	Public awareness of marine environmental quality and its relationship for policy support on marine waste management.	Marine pollution bulletin	3. Study objectives not relevant
Miller SA 2020	Five Misperceptions Surrounding the Environmental Impacts of Single-Use Plastic.	Environmental science & technology	3. Study objectives not relevant
Rambonnet L 2019	Making citizen science count: Best practices and challenges of citizen science projects on plastics in aquatic environments.	Marine pollution bulletin	3. Study objectives not relevant
Ricke IJ 2022	Knowledge, Attitudes, and Behaviors Regarding Chemical Exposure among a Population Sample of Reproductive-Aged Women	International journal of environmental research and public health	3. Study objectives not relevant
Rist S 2018	A critical perspective on early communications concerning human health aspects of microplastics.	The Science of the total environment	3. Study objectives not relevant
Wee SY 2024	Public perception on human exposure risk: A case study on endocrine disrupting compounds in the environment	Ecotoxicol. Environ. Saf.	3. Study objectives not relevant
Locritani M 2019	Assessing the citizen science approach as tool to increase awareness on the marine litter problem.	Marine pollution bulletin	4. Evaluating the effectiveness of an intervention on knowledge and behavior
Skoric MM 2022	Reducing the Use of Disposable Plastics through Public Engagement Campaigns: An Experimental Study of the Effectiveness of Message Appeals, Modalities, and Sources.	International journal of environmental research and public health	4. Evaluating the effectiveness of an intervention on knowledge and behavior
