# Supplementary material for: Surveys of Knowledge and Awareness of Plastic Pollution and Risk Reduction Behavior in the General Population: A Systematic Review
Source: Int J Environ Res Public Health. 2025 Jan 27;22(2):177. doi: 10.3390/ijerph22020177 (PMC11855307; doi:10.3390/ijerph22020177)
Supplement: Supplementary file 1 [file ijerph-22-00177-s001.zip › Supplementary material/Table S5.pdf]

Table S5: Thematic areas addressed by the included studies

Reference number	First author	Year of publication	Was the questionnaire provided in its entirety (as an electronic appendix or in print)?	Thematic areas		
				Levels of knowledge about different types of plastics	Levels on knowledge about the risks associated with plastics	Awareness of actions to reduce possible harms from plastic pollution
38	Miguel I	2024	No		Perceptions of plastic pollution in different environmental compartments	Recycling attitudes and behaviors
28	Dagiliūt R	2023	No			Self-stated pro-environmental behaviour related to plastic use and handling
35	Garcia-Vazquez E	2022	Yes	Microplastics, microplastics sources, environmental sites of accumulation	Ways in which microplastics can enter the human body	Actual behavior of checking for microplastics and intentions to adopt pro-environmental behaviors
39	Oleksiuk K	2022	No	Microplastics, their source presence in parts of the environment, water and food	Microplastics metabolism in the living organism, and possible health hazards	Possible ways of reducing the amount of microplastic exposure.
36	Li Y	2022	Yes			Pro-environmental behaviors
33	Filho WL	2022	Yes	Bioplastics, bio-based and biodegradable products	Bioplastics impact on human health	Usage and buying of bioplastics

32	Filho WL	2021	No	Characteristics of bioplastics and availability in respondents' countries	Effects of plastic pollution	Efforts to reduce plastic consumptions
40	Soares J	2021	No		The perception regarding the threats that plastic pollution may pose	Pro-environmental behaviours: frequency, reasons for non-enactment
27	Charitou A	2021	No		Knowledge on marine plastic pollution	Attitudes of the participants including willingness to pay and intention to act against plastic pollution
34	Forleo MB	2021	No	Marine and beach litter, different types of plastics, microplastics	Sources and impacts of plastic litter in the marine waters and on the beach	Behavior towards plastics
37	Menzel C	2021	Yes		Valence and risk evaluations of the three plastic forms in comparison to other material and other risks	
41	Thiele CJ	2021	Yes		Concern about microplastics in relation to other environmental issues and reasons; perception of hazardousness of microplastics, and differences between lay-people and experts	

25	Barbir J	2021	No		Impacts of plastics on human health	Efforts to reduce use of plastic and willingness to change current practices and habits
26	Cammalleri V	2020	No		Level of knowledge on the theme "Microplastics pollution"	
29	Deng L	2020	Yes	The public's perception and attitude towards microplastics		Behavioral preferences for microplastics, willingness to reduce emissions, and policy recommendations for reducing emissions.
31	Dilkes-Hoffman L	2019b	Yes	What bioplastics are and their characteristics	Environmental impact and utility	End-of-life management
30	Dilkes-Hoffman L	2019a	Yes		Plastics as a serious environmental issue. Association of plastics with food packaging, convenience and environmental concern	Action for reducing plastic use. Responsibility for reducing plastic use

Note: The classification into themes was based solely on the terminology used in the articles, since the full questionnaire was available for fewer than half of the examined studies
