# Supplementary material for: Surveys of Knowledge and Awareness of Plastic Pollution and Risk Reduction Behavior in the General Population: A Systematic Review
Source: Int J Environ Res Public Health. 2025 Jan 27;22(2):177. doi: 10.3390/ijerph22020177 (PMC11855307; doi:10.3390/ijerph22020177)
Supplement: Supplementary file 1 [file ijerph-22-00177-s001.zip › Supplementary material/Table S7.pdf]

Table S7: Quality assessment of included studies using AXIS

	Introduction	Methods								
Author (Year of pubblication)	Were the aims/objectives of the study clear?	Was the study design appropriate for the stated aim(s)?	Was the sample size justified?	Was the target/ reference population clearly defined? (Is it clear who the research was about?)	Was the sample frame taken from an appropriate population base so that it closely represented the target/reference population under investigation?	Was the selection process likely to select subjects/participants that were representative of the target/ reference population under investigation?	Were measures undertaken to address and categorize non-responders?	Were the risk factors and outcome variables measured appropriate to the aims of the study?	Were the risk factors and outcome variables measured correctly using instruments/ measurements that had been trialed, piloted or published previously?	Is it clear what was used to determined statistical significance and/or precision estimates? (e.g., p values,CIs)	Were the methods (including statistical methods) sufficiently described to enable them to be repeated?
Miguel I (2024)	Y	N	N	N	CT	CT	N	N	N	N	N
Dagiliūt R (2023)	Y	N	N	Y	CT	CT	N	Y	Y	Y	CT
Garcia-Vazquez E (2022)	Y	N	N	Y	CT	CT	N	Y	Y	Y	Y
Oleksiuk (2022)	N	N	Y	Y	N	N	N	Y	N	Y	Y
Li (2022)	Y	N	N	N	N	N	N	Y	N	N	N
Filho (2022)	Y	N	N	N	N	N	N	Y	N	Y	Y
Filho (2021)	Y	N	N	N	N	N	N	Y	N	Y	Y
Soares (2021)	Y	N	N	N	N	N	N	Y	N	Y	Y
Charitou (2021)	Y	N	N	Y	N	N	N	Y	N	Y	Y
Forleo (2021)	Y	N	N	N	N	N	N	Y	Y	Y	Y
Menzel 2021	N	N	Y	N	N	N	N	CT	N	Y	Y
Thiele (2021)	Y	N	N	N	N	N	N	Y	N	N	N
Barbir (2021)	Y	N	N	Y	N	N	N	Y	N	Y	Y
Cammalleri (2020)	Y	N	N	Y	Y	CT	N	Y	N	Y	Y
Deng (2020)	Y	N	N	Y	N	CT	N	Y	Y	Y	Y
Dilkes-Hoffman (2019b)	Y	Y	N	Y	Y	Y	N	Y	Y	Y	Y
Dilkes-Hoffman (2019a)	Y	Y	N	Y	Y	Y	N	Y	Y	Y	Y

Note: Y = Yes, N = No, CT = Cannot Tell

Table S7 (Continued)

	Results					Discussion		Others	
Author (Year of pubblication)	Were the basic data adequately described?	Does the response rate raise concerns about non-response bias?	If appropriate, was information about non-responders described?	Were the results internally consistent?	Were the results for the analyses described in the methods, presented?	Were the authors' discussions and conclusions justified by the results?	Were the limitations of the study discussed?	Were there any funding sources or conflicts of interest that may affect the authors' interpretation of the results?	Was ethical approval or consent of participants attained?
Miguel I (2024)	N	CT	N	Y	Y	Y	Y	N	Y
Dagiliūt R (2023)	Y	CT	N	Y	Y	Y	Y	N	CT
Garcia-Vazquez E (2022)	N	CT	N	Y	Y	Y	Y	N	Y
Oleksiuk (2022)	Y	CT	N	Y	Y	Y	Y	N	Y
Li (2022)	Y	CT	N	Y	N	N	Y	N	Y
Filho (2022)	Y	CT	N	Y	Y	N	Y	N	N
Filho (2021)	Y	CT	N	Y	Y	N	Y	CT	N
Soares (2021)	N	CT	N	Y	Y	Y	Y	N	Y
Charitou (2021)	N	CT	N	Y	Y	N	N	N	N
Forleo (2021)	Y	CT	N	Y	Y	N	Y	N	CT
Menzel (2021)	N	CT	N	CT	Y	N	Y	CT	Y
Thiele (2021)	N	N	N	Y	Y	N	Y	N	Y
Barbir (2021)	N	CT	N	Y	Y	N	N	N	N
Cammalleri (2020)	Y	CT	N	Y	Y	Y	Y	CT	CT
Deng (2020)	Y	CT	N	Y	Y	N	Y	N	Y
Dilkes-Hoffman (2019b)	Y	CT	N	Y	Y	Y	N	CT	CT
Dilkes-Hoffman (2019a)	Y	CT	N	Y	Y	Y	N	CT	CT

Note:

- Methodological quality assessment of the 17 included studies using the Appraisal Tool for Cross-Sectional Studies (AXIS) [Downes et al. doi: 10.1136/bmjopen-2016-011458].
- Y = Yes, N = No, CT = Cannot Tell
